# Ultra-fast direct growth of metallic micro- and nano-structures by focused ion beam irradiation

**DOI:** 10.1038/s41598-019-50411-w

**Published:** 2019-10-01

**Authors:** Rosa Córdoba, Pablo Orús, Stefan Strohauer, Teobaldo E. Torres, José María De Teresa

**Affiliations:** 1 Instituto de Ciencia de Materiales de Aragón (ICMA), Universidad de Zaragoza-CSIC, 50009 Zaragoza, Spain; 2 Departamento de Física de la Materia Condensada, Universidad de Zaragoza, 50009 Zaragoza, Spain; 3 Laboratorio de Microscopías Avanzadas (LMA) - Instituto de Nanociencia de Aragón (INA), Universidad de Zaragoza, 50018 Zaragoza, Spain; 4 Present Address: Instituto de Ciencia Molecular, Universitat de València, Paterna, València, 46980 Spain; 5 Present Address: Instituto de Nanociencia y Nanotecnología CNEA-CONICET, Centro Atómico Bariloche, Av. Bustillo 9500, 8400 San Carlos de Bariloche, Argentina; 6 Present Address: Walter Schottky Institute and Physics Department, Technical University of Munich, Am Coulombwall 4, D-85748 Garching, Germany

**Keywords:** Nanowires, Electronic devices, Electronic properties and materials

## Abstract

An ultra-fast method to directly grow metallic micro- and nano-structures is introduced. It relies on a Focused Ion Beam (FIB) and a condensed layer of suitable precursor material formed on the substrate under cryogenic conditions. The technique implies cooling the substrate below the condensation temperature of the gaseous precursor material, subsequently irradiating with ions according to the wanted pattern, and posteriorly heating the substrate above the condensation temperature. Here, using W(CO)_6_ as the precursor material, a Ga^+^ FIB, and a substrate temperature of −100 °C, W-C metallic layers and nanowires with resolution down to 38 nm have been grown by Cryogenic Focused Ion Beam Induced Deposition (Cryo-FIBID). The most important advantages of Cryo-FIBID are the fast growth rate (about 600 times higher than conventional FIBID with the precursor material in gas phase) and the low ion irradiation dose required (∼50 μC/cm^2^), which gives rise to very low Ga concentrations in the grown material and in the substrate (≤0.2%). Electrical measurements indicate that W-C layers and nanowires grown by Cryo-FIBID exhibit metallic resistivity. These features pave the way for the use of Cryo-FIBID in various applications in micro- and nano-lithography such as circuit editing, photomask repair, hard masks, and the growth of nanowires and contacts. As a proof of concept, we show the use of Cryo-FIBID to grow metallic contacts on a Pt-C nanowire and investigate its transport properties. The contacts have been grown in less than one minute, which is considerably faster than the time needed to grow the same contacts with conventional FIBID, around 10 hours.

## Introduction

Nanotechnology based on top-down approaches relies on the use of nanofabrication techniques to create nano-patterns and build nano-devices. The most prominent example is the use of optical lithography for the fabrication of integrated circuits. In this technology, the design of a photo-mask is transferred to a photo-resist, then to the material of interest. In the semiconductor industry, the most advanced ultraviolet (UV) optical lithography techniques are utilized to achieve progress in each new node of microelectronic devices, with present-day devices being available on 300 mm wafers with critical dimensions around 10 nm. The 7 nm node, which makes use of Extreme UV optical lithography, is foreseen for 2020^1^. Other nanolithography techniques such as nanoimprinting^2^ and scanning-probe lithography^3^ are under development for niche applications. Moreover, the use of focused beams of charged particles for nanopatterning is widespread. Thus, Electron Beam Lithography (EBL), based on the usage of a Focused Electron Beam (FEB) and an electron-sensitive resist, is very popular for lab-scale applications given its affordable cost and high resolution (sub-10 nm patterning is feasible)^4^. Focused Ion Beam (FIB) milling is another successful technology for nanopatterning^5,6^. Given the higher weight of ions compared to electrons, a focused beam of accelerated ions (Ga^+^, Ne^+^, He^+^…) provides a high linear momentum with the capability to remove material locally. As FIB milling does not require masks and provides high resolution, it has become a broadly-used technique for device prototyping^7^, circuit edit^8^ and mask repair^9^.

The technology of focused beams of charged particles can be combined with precursor materials to grow deposits locally^10–12^. In this technique, the precursor molecules in gas phase are delivered onto the substrate surface by means of a nearby gas-injection system whilst the FEB or the FIB is scanned over the substrate, inducing the growth of a deposit shaped as the pattern traced by the beam. In the so-called Focused Electron Beam Induced Deposition (FEBID) and Focused Ion Beam Induced Deposition (FIBID) techniques, the attained lateral resolution of the deposits is regularly within a few tens of nm, but proof-of-concept experiments have shown resolution as good as 3 nm for the growth of Pt-based dots by FEBID^13^, and 10 nm for the growth of cobalt lines by He^+^-FIBID^14^. FEBID/FIBID techniques have found applications for the local growth of metal lines used to establish electrical connection between different parts of microelectronic circuits during circuit edit^15,16^, for the restoration of material continuity when repairing defects found in optical masks^17,18^, and, more recently, for the growth of functional materials in the fields of magnetism^19,20^, superconductivity^21^, nano-devices^22^, nano-optics and plasmonics^23,24^, and sensing^25^. In these applications, FEBID and FIBID bring in a high lateral resolution, the capability for three-dimensional growth, the functionality of the deposited material, the tunability of the deposit composition during growth or by post-processing, and the freedom in the choice of substrate. However, a more extensive use of FEBID/FIBID is hampered by a serious limitation: *the low throughput attainable in the standard FEBID/FIBID process*, *which relies on precursors dissociated in the gas phase*. In the following, we focus our attention to one particularly important application of FEBID/FIBID: the growth of metallic interconnects, of special relevance for circuit edit (which is a crucial step during the development of new integrated circuits), and for placing metal contacts at the micro- and nano-scale.

In the growth of nanoscale metallic interconnects, a high growth rate and a low electrical resistivity are preferred. In the Supplementary File, the growth rate is represented as a function of the resistivity for typical FEBID and FIBID deposits used in metallic interconnects, as obtained from existing references (see Fig. S1). One can notice that FIBID processes are faster than FEBID (typically about three orders of magnitude) and give rise to lower resistivity (by several orders of magnitude). FIBID is thus preferred to FEBID to produce metal connections. W-based deposits by FIBID using the W(CO)_6_ precursor are broadly used in this application given the low electrical resistivity achievable, generally in the range of 100–500 μΩcm^16,21,26–28^. The deposits typically contain (in atomic %) around 40% of W, 40% of C, 10% of O and 10% of Ga, so they are termed W-C deposits^29^. The value of the electrical resistivity in these W-C deposits depends on the specific growth parameters, which determine the composition and microstructure (W-C deposits grown by FEBID show higher electrical resistivity caused by a lower metallic content)^30^. FIBID deposits generally show a higher metallic content than their FEBID counterparts, which stems from a more efficient precursor dissociation. The growth rate of W-C deposits by FIBID shows values around 0.1 μm^3^/nC^21,26,28^. This is certainly a time-consuming process that would benefit from further developments enhancing the growth rate. As an example, the growth of one metal line with dimensions of 10 μm length, 1 μm width and 30 nm thickness using a Ga^+^ ion beam current of 10 pA requires ∼5 minutes.

The main reason for the low growth rate is that the precursor molecules are in gas phase and form a single (sub)monolayer of adsorbed molecules on the substrate surface. The most efficient mechanism for precursor dissociation needs secondary electrons with a kinetic energy not far from the energy required to break molecule bonds, in the range of a few eV^31^. Once a precursor molecule has been dissociated, a new molecule has to diffuse and occupy its place, which is a slow process. The adsorbed molecules also have a finite probability of becoming detached from the substrate. Taking into account these effects, FIBID with precursors in gas phase presents serious limitations in speed. If the values are translated into an area dose, for thicknesses in the range 10–100 nm they fall in the range of 10^3^–10^5^ μC/cm^2^, higher than typical irradiation doses used in EBL (<10^3^ μC/cm^2^)^32^. This entails long processing times and can lead to the appearance of side effects in the deposit and in the substrate: ion-induced defects, implantation, amorphization, milling, etc^33,34^. Regarding the side effects on the deposit itself, whereas an excess Ga^+^ exposure can be beneficial to increase the overall metallic content of the deposit, thus decreasing its electrical resistivity, it can also be detrimental for deposits aiming for magnetic or plasmonic functionality. Thus, an excess Ga^+^ exposure would lead in the first case to deposits with lower magnetic content, and, in the second case, to deposits with lower plasmonic activity. As for the side effects on the substrate, an excess Ga^+^ exposure can lead to unwanted modifications of its original properties such as the substrate’s electrical conductivity and optical properties, its crystalline quality, grain size, thickness, etc. In summary, new technical improvements in FIBID towards the decrease of the required ion doses would have tremendous beneficial impact on two key parameters: *the decrease of the processing time and of potentially-detrimental side effects caused by the ion irradiation*.

**Figure 1:**
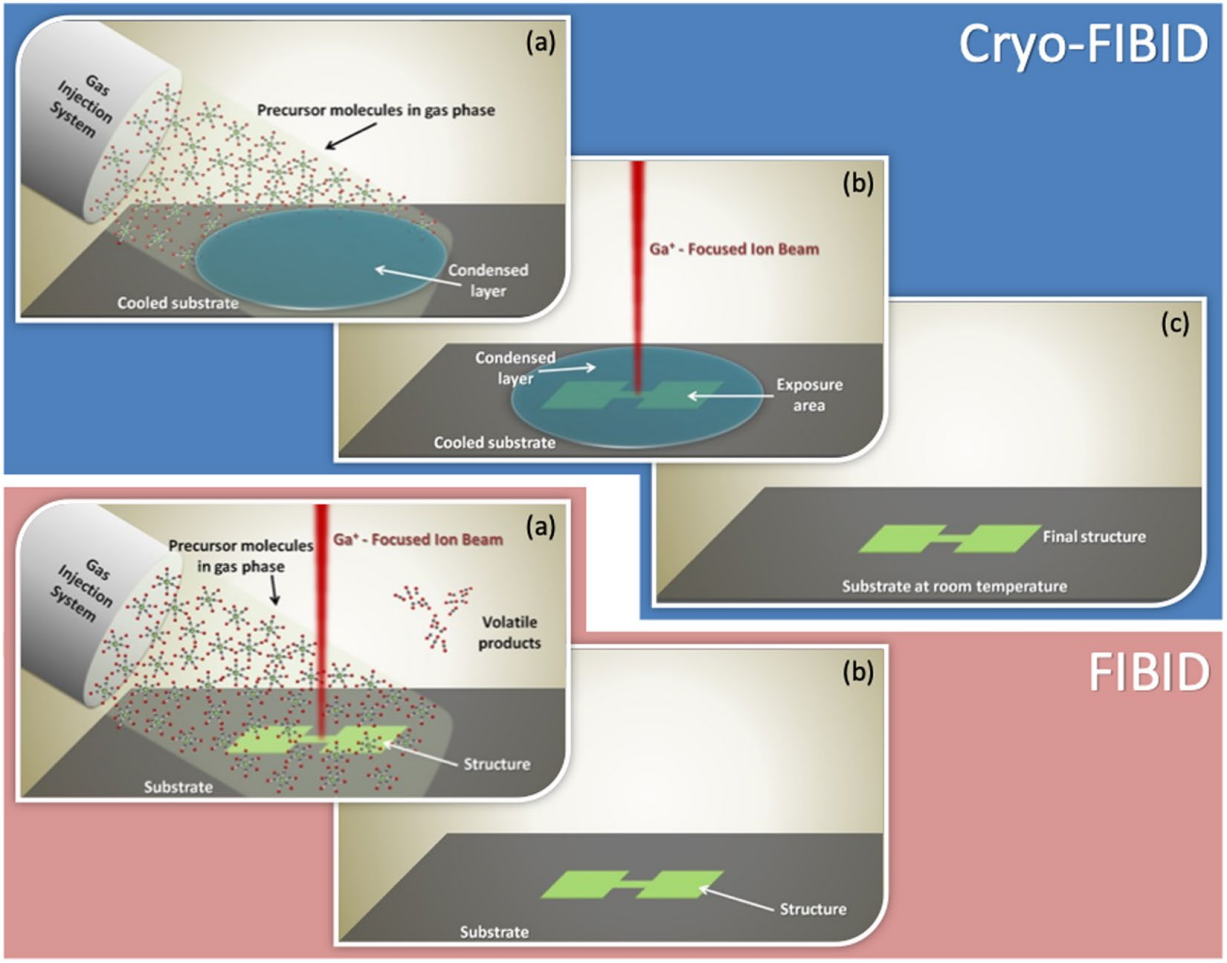
Comparison of FIBID steps using precursor molecules either in the gas phase (standard FIBID) or forming a condensed layer (Cryo-FIBID). In the Cryo-FIBID process, the substrate is first cooled below the precursor condensation temperature. Then, the gaseous precursor is delivered through the gas injection system for a short time (typically a few seconds) to produce a thin layer of condensed precursor over the substrate surface. After FIB exposure of the corresponding areas, the substrate is heated to room temperature and the material remains only at the exposed areas.

In the following, we show a technical solution to decrease the required ion dose in FIBID, which hereafter will be called *Cryo-FIBID*. This new strategy is based on obtaining a condensed layer of precursor molecules by cooling the substrate below the precursor condensation temperature. Then, the ion dose needed to produce a deposit of a given thickness is almost three orders of magnitude below that required to grow a deposit of the same thickness using the precursor in gas phase. After ion beam exposure, heating the substrate above the precursor condensation temperature reveals a deposit shaped as the pattern scanned by the beam. In Fig. 1, the steps of both FIBID processes, the standard one with the precursor in the gas phase and the new one with the precursor in the condensed phase, are sketched. In the Supplementary File, a video animation (Video S1) is included to facilitate the understanding of this novel nanofabrication technique. To our understanding, this is the first time that a focused ion beam is used to create a *metal deposit with nanoscale dimensions by means of condensed precursor layers*. Previous work using cryogenic conditions has shown the potential of Ar^+^ broad beams to create Sn metal deposits^35^ and focused electron beams to grow non-metallic Pt deposits^36,37^. On the other hand, the use of water ice and organic ice resists using cryogenic conditions has been proposed in EBL, but it requires high electron doses, as well as subsequent metal evaporation^38,39^. Our experimental results show that Cryo-FIBID using the W(CO)_6_ precursor is a viable technique to obtain metal layers and nanowires with ultra-fast growth rate and minimal ion irradiation. By avoiding the two main disadvantages of FIBID with precursor molecules in gas phase, the low processing speed and the side effects of a high ion irradiation, Cryo-FIBID holds great potential for those applications in nanotechnology that require the growth of metallic layers, nanowires, and contacts.

## Results

### Formation of a homogeneous W(CO)_6_ condensed layer with controlled thickness

The first step of Cryo-FIBID requires the formation of a homogeneous layer of condensed W(CO)_6_ with controlled thickness. We have carried out processes opening the injector valve for 10 s and with distances between the gas injector end and the substrate of 1 mm, 5 mm and 10 mm. These conditions lead to condensed precursor layers at the FIB incidence point with respective thicknesses around 6000 nm, 400 nm and 30 nm. Taking into account that the Ga^+^ penetration depth at 30 kV on silicon is around 30 nm, the value of 10 mm working distance is preferred here. At 10 mm working distance, a homogeneous W(CO)_6_ precursor condensed layer is obtained when the temperature of the substrate is −100 °C (see Fig. S2 in the Supplementary File), whereas, at higher temperatures, the condensed layer contains some voids and displays higher surface roughness. In Fig. S3 in the Supplementary File, SEM micrographs of the condensed layer at 5 mm working distance are shown, indicating a more inhomogeneous behaviour of the condensed layer.

### Influence of the irradiation dose on the deposit compactness and composition

After the optimization of the injector-substrate distance (10 mm) and of the substrate working temperature (−100 °C), experiments using various Ga^+^ doses were carried out to obtain deposits with high compactness. The obtained results indicate that a very low Ga^+^ dose is sufficient to expose the W(CO)_6_ condensed layer, giving rise to a compact deposit. SEM micrographs of cryo-deposits grown using two different irradiation doses, one of them close to the optimal dose, are compared in Fig. S4 in the Supplementary File. Whereas the deposit exposed with 35.7 μC/cm^2^ Ga^+^ dose is compact and void-free, the deposit exposed with 4.21 μC/cm^2^ Ga^+^ dose is not compact and shows a high void density. The Ga^+^ dose of 35.7 μC/cm^2^ is almost a factor of 1000 lower than the dose required to obtain the same deposit with the precursor in gas phase considering a growth rate of 0.1 μm^3^/nC. This effect is ascribed to the large amount of precursor molecules available for dissociation when the precursor is in the condensed state (layer of 30 nm thickness), contrary to the case of standard FIBID, where only a precursor (sub)monolayer adsorbed on the substrate’s surface can be dissociated.

### Composition study by transmission electron microscopy

**Figure 2:**
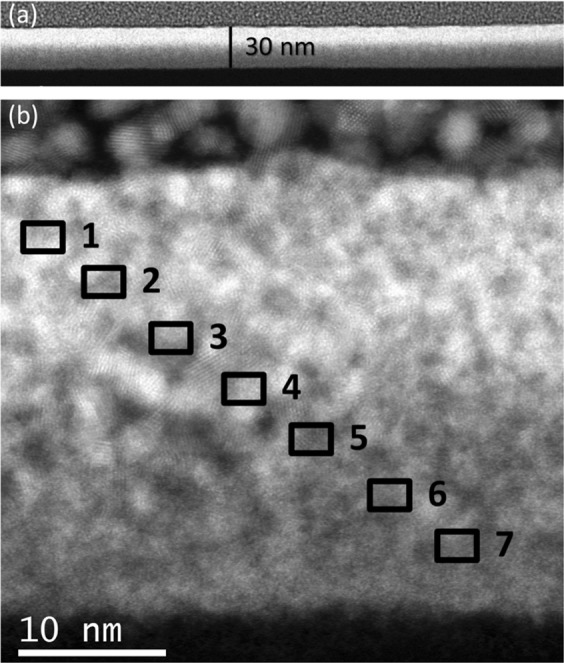
Low-resolution (**a**) and high-resolution (**b**) STEM images of a W-C cryo-deposit grown under optimized conditions, as discussed in the text. The black squares pinpoint the areas where EDS experiments have been carried out to investigate the composition along the deposit thickness.

Once the good efficiency of the Cryo-FIBID process has been established, the composition of the deposit has been studied to determine its suitability to act as a metal interconnect. For that, lamellae have been extracted from a sample fabricated under a Ga^+^ dose of 55 μC/cm^2^ and studied by Scanning Transmission Electron Microscopy (STEM). As shown in Fig. 2(a), the deposit exhibits homogenous thickness (∼30 nm), low roughness and absence of voids. The deposit looks darker at the bottom part and brighter towards the top, which suggests an evolution of the composition with thickness. The composition has been investigated by Energy-Dispersive X-ray Spectroscopy (EDS) in selected areas from top to bottom, as marked with black squares in Fig. 2(b). The obtained data are shown in Fig. S5 in the Supplementary File. In brief, at the top half part of the deposit, the W atomic content is around 22%, decreasing below 10% at the bottom half part. The rest of the deposit contains C and O, which are elements present in the precursor molecule. As expected, Ga is generally absent in the deposit within the detection limits of the EDS technique.

### Electrical characterization

**Figure 3:**
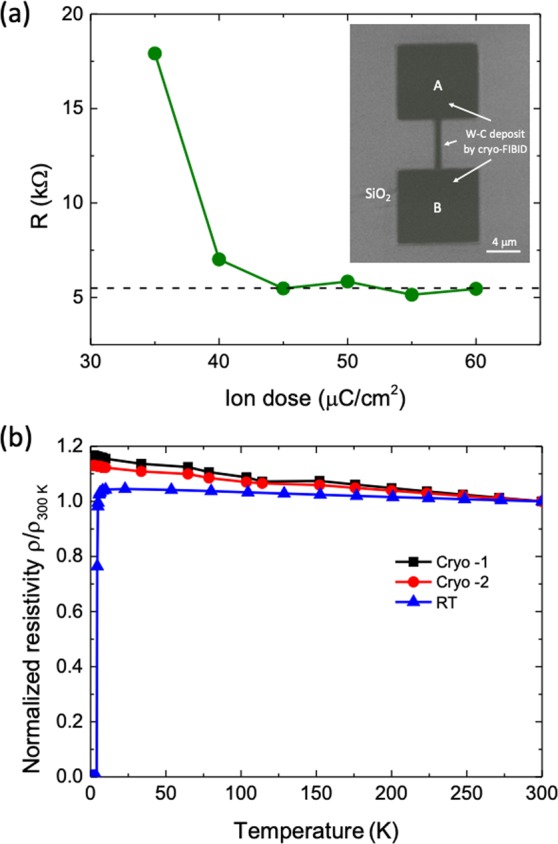
(**a**) Electrical resistance at room temperature of W-C cryo-deposits as a function of the Ga^+^ dose used for their fabrication. The inset shows a colored image of one of the devices measured; (**b**) Electrical measurements as a function of temperature of W-C cryo-deposits (Cryo-1, Cryo-2) and a W-C FIBID deposit grown with precursor in the gas phase at room temperature (RT). The room-temperature resistivity of the W-C cryo-deposits is ~800 μΩcm, whereas that of the W-C FIBID deposits grown with precursor in the gas phase is ~300 μΩcm.

In order to investigate the metallic character of the deposits and to optimize the Ga^+^ dose with respect to the resistivity, electrical measurements were first performed at room temperature by the two-probe technique using electrical microprobes. In a second step, deposits grown under optimized conditions were electrically characterized from room temperature down to 2 K by four-probe measurements (using Pt-FIBID electrical contacts). The experiments performed with the electrical microprobes were carried out using the sample geometry shown in Fig. 3(a). Given that a Ga^+^ dose of 35.7 μC/cm^2^ produces a compact deposit, the experiments were conducted on samples with a Ga^+^ dose ranging from 35 μC/cm^2^ up to 60 μC/cm^2^. Current-versus-voltage (I-V) experiments were performed on the fabricated structures, showing a linear dependence as expected for a metallic behaviour (see Fig. S6 in the Supplementary File). From linear fits to the I-V data, the electrical resistance was extracted. The results obtained in samples grown under ion doses from 35 to 60 μC/cm^2^ are displayed in Fig. 3(a). This graph indicates that the resistance decreases as a function of the dose until 45 μC/cm^2^ and then saturates to the value of ∼5.5 kΩ for higher doses. From this result, it can be inferred that optimum low-resistance W-C cryo-deposits are obtained by using a Ga^+^ dose within the interval 45 μC/cm^2^ to 60 μC/cm^2^.

The electrical measurements as a function of temperature were carried out on samples with a Ga^+^ dose of 55 μC/cm^2^. As shown in Fig. 3(b), the resistivity does not vary much with decreasing temperature, undergoing a slight increase of 15% with respect to the value at room temperature. This behaviour is reminiscent of the temperature-dependent behaviour of the resistivity of W-C FIBID deposits grown using standard conditions with the precursor in gas phase, also shown in Fig. 3 for the sake of comparison. Following similar data analysis to that performed in other materials^40,41^, the detailed analysis of the resistance with temperature confirms the metallic character of the W-C cryo-FIBID deposits (see Fig. S7 in the Supplementary File). The room-temperature resistivity values of the two cryo-deposits shown in Fig. 3(b), obtained taking into account the resistance and the deposit dimensions, are 800 μΩcm and 940 μΩcm, being slightly higher than the ones grown at room temperature using standard FIBID. It is also worth mentioning that the studied W-C cryo-deposits do not become superconducting down to 2 K, in contrast with the W-C FIBID deposits grown at room temperature, which become superconducting at ~5 K^28^. Although the origin of superconductivity in W-C deposits is still an open question, an obvious difference between deposits grown by both methods is the composition, with higher W and Ga content in the superconducting ones.

### Achievement of high resolution in patterning by Cryo-FIBID

**Figure 4:**
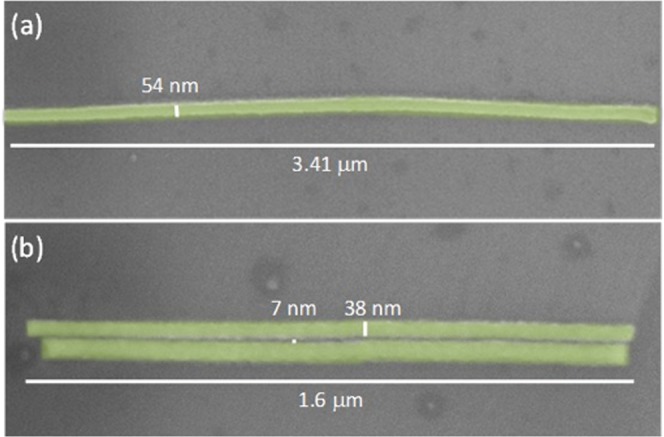
Artificially-coloured SEM micrographs of W-C cryo-deposits grown using single-pixel-line patterning during Ga^+^ exposure. (**a**) isolated 54 nm-wide line; (**b**) two nearby 38 nm-wide lines, separated by 7 nm.

The results shown in the previous sections indicate that W-C cryo-deposits are promising for the ultra-fast growth of metallic interconnects. In order to probe their applicability to nanotechnology, we have investigated the limitation in the achievable lateral resolution. Single-pixel-line patterning using low ion current (1 pA) was performed in the Ga^+^ exposure step in order to obtain structures as narrow as possible. SEM micrographs of some of the achieved nanostructures are presented in Fig. 4. As shown in Fig. 4(a), 54 nm-wide nanowires with an aspect ratio higher than 60 have been fabricated using an 80 μC/cm^2^ dose. In addition, closely-spaced lines have been grown using a 60 μC/cm^2^ dose, as shown in Fig. 4(b). Two 38 nm-wide nanowires separated by only 7 nm is a remarkable result. W-C Ga^+^-FIBID deposits grown using standard conditions with the precursor in gas phase have lateral resolution around 50 nm and, due to proximity effects, two lines should be separated at least 60 nm to be resolved^42^. Reasons for this outstanding behaviour of W-C cryo-deposits will be discussed in the next section.

## Discussion

The set of results shown in previous sections suggests the great potential of Cryo-FIBID for applications in nanotechnology. In the following, we discuss open scientific and technological challenges raised by these results. A first point of discussion concerns the precursor condensation dynamics. Our results indicate a strong dependence of the homogeneity and roughness of the condensed layer with the distance between the precursor injector and the substrate as well as with the substrate temperature, underlining the importance of the dynamical processes behind the physical phenomenon of condensation. In addition, a spatial dependence of the condensed layer thickness with the injector’s dimensions and its angle and distance with respect to the substrate is expected and should be taken into account^43,44^. A better knowledge of these aspects would facilitate a more efficient extrapolation of the present results to other designs of injectors and to the use of other precursors for Cryo-FIBID.

A second point of interest relates to the Ga^+^-induced mechanisms triggering the precursor dissociation in the condensed state of the W(CO)_6_ precursor. To the best of our knowledge, this subject has not been addressed theoretically yet, but it would be helpful towards future optimization of Cryo-FIBID in terms of the process speed and the metal content. Nevertheless, some hints can be anticipated according to related studies. In FIBID growth, precursor dissociation is induced by the primary ions, the recoil atoms and the generated secondary electrons^31^. The relative relevance of each of these processes depends to a large extent on the mass and ionic state of the primary ions, the specific precursor molecule and the underlying substrate. On top of these deposition processes, FIB milling is competing and can alter the sample geometry^45^. Although the growth of Ga^+^-FIBID deposits with the W(CO)_6_ precursor in gas phase has been explained by precursor dissociation caused by secondary electrons^46^, the situation could be different when the precursor is in the condensed phase, which should be specifically addressed. In particular, it is of great interest to understand the small proximity effect observed in Fig. 4(b), with two nanowires separated by only 7 nm. This result suggests that, in this case, the main mechanism for precursor deposition is ion-triggered, thus localized not far from the primary-ion impact point. Therefore, Cryo-FIBID using Ga^+^ ion sources holds tremendous potential for the fabrication of highly-dense nanostructures, in sharp contrast to the use of the precursor in gas phase, which is accompanied by a large proximity effect due to deposition induced by secondary electrons^42^. Furthermore, one could even think of the application of Cryo-FIBID to grow materials acting as hard masks for ultra-high-resolution lithography, as previously done using FEBID materials^47^, but requiring a charge dose orders of magnitude below.

**Figure 5:**
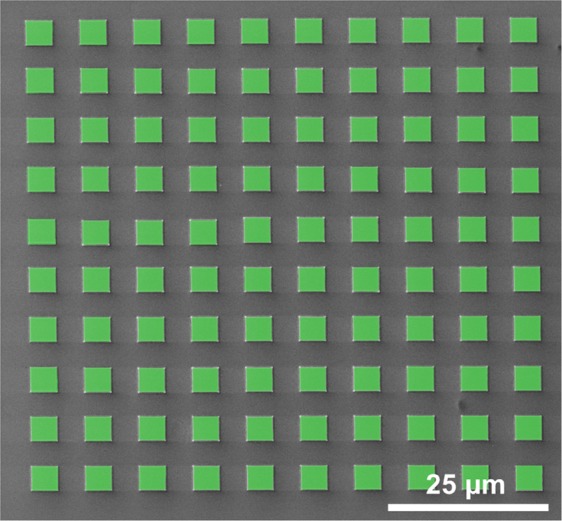
Artificially-coloured SEM micrograph of a W-C Cryo-deposit array, composed of 100 rectangles of size 4 μm × 3.85 μm, grown in a single Ga^+^- irradiation exposure. The total ion irradiation time was only 85 seconds, to be compared with more than 14 hours if the standard FIBID process at room temperature had been used.

The obtained results are outstanding in terms of the small charge dose needed to grow nanoscale metal structures, around 50 μC/cm^2^. Thus, Cryo-FIBID excels popular nanolithography techniques such as EBL, which typically requires electron doses one order of magnitude higher^32,48^. An experiment demonstrating the scalability of the technique has been performed. For that, an array of 100 rectangles of size 4 μm × 3.85 μm was fabricated with a single Ga^+^- irradiation exposure of 85 seconds. An SEM micrograph of the fabricated array is shown in Fig. 5. One can calculate how long it would take to grow a W-C layer of area 1 × 1 mm^2^, amounting to 15 hours. This is competitive with respect to other common nanolithography techniques like EBL with PMMA resist, which uses higher beam current (nA regime) but requires higher irradiation dose, and additional steps for resist coating, resist development, metal evaporation and lift-off^32^.

## Outlook

Given the short processing time, the high resolution and the lower irradiation dose needed, W-C Cryo-FIBID could be of great interest for circuit editing in semiconductor industry^15,16^. By the tremendous enhancement of the growth rate of metallic W-C deposits used for circuit rewiring, the total processing time for editing a circuit would be considerably shortened, leading to significant time and cost reduction. As an example, exposure of one metal line with dimensions of 10 μm length, 1 μm width and 30 nm thickness would only take 0.5 s using a Ga^+^ ion current of 10 pA, in sharp contrast to ~5 minutes (600 times higher) if the precursor is in gas phase (growth rate of ~0.1 μm^3^/nC). Similarly, Cryo-FIBID holds great potential for mask repair in optical lithography. Nowadays, FIBID competes with FEBID for growth of restoring materials during mask repair^17,18^. The main disadvantage of FIBID for this application, which is the ion damage that changes the mask optical properties, could be avoided by means of Cryo-FIBID given the very low ion dose needed. Furthermore, the growth rate of Cryo-FIBID compared to FEBID is another great advantage for this application.

**Figure 6:**
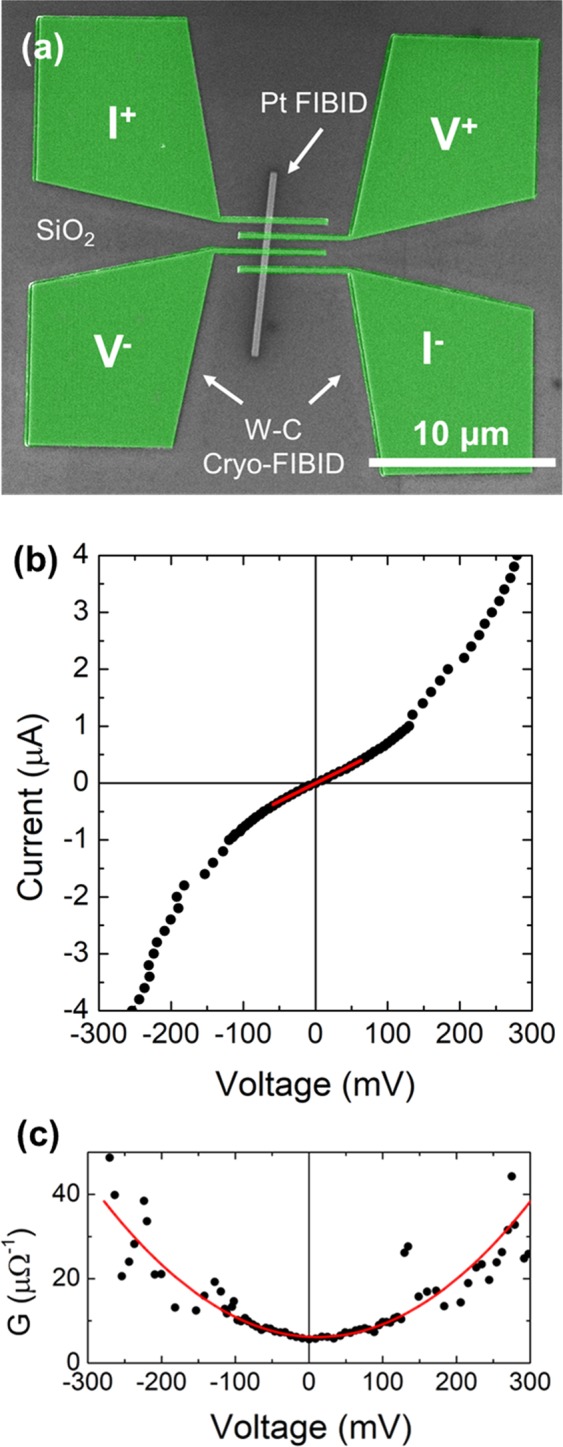
(**a**) Artificially-coloured SEM micrograph of the measured Pt-C nanowire with the four electrical contacts grown by W-C Cryo-FIBID; (**b**) Current-voltage dependence of the Pt-C nanowire, with a linear fit (red line) at low currents indicating a resistivity of 9 × 10^4^ μΩ·cm; (**c**) Differential conductance G(V) = dI/dV, obtained by numerical differentiation of the results shown in (**b**) and visual guideline (red colour).

On the other hand, a great potential exists for W-C Cryo-FIBID to directly grow metallic contacts on nanowires, micro/nano-particles, thin films of topological insulators and flakes/films of 2D materials, with two key advantages: time saving and minimized ion-induced damage. In general, the EBL technique is used to grow metal contacts on these materials. However, resist residues are often unavoidable and alter the electrical properties^49^, and the resist cannot be dispensed on non-flat substrates needed in some cases, such as cantilevers. This is why direct growth of metal contacts by FIBID has been used in various instances despite the perturbing effects of Ga implantation and induced damage^50^. Such problems are eliminated to a large extent by the use of W-C Cryo-FIBID, given the much lower ion dose needed. As a proof of concept, we have used W-C Cryo-FIBID to grow electrical contacts onto a 25 nm-thick Pt-C nanowire, as shown in Fig. 6(a). The electrical resistance of such Pt-C nanowire has been studied in the past using standard electrical contacts^41^, serving as a good reference to test the potential of Cryo-FIBID for this application. Four-probe current-versus-voltage measurements are shown in Fig. 6(b,c), indicating a slightly non-linear behaviour, as expected for a nanowire with such thickness^41^. The nanowire’s resistivity, 9 × 10^4^ μΩcm, agrees with previous measurements using standard electrical contacts^41^. The W-C Cryo-FIBID structure shown in Fig. 6(a) has required only 50 s of ion irradiation, a strikingly shorter time than the 10-hour ion irradiation needed if the same structure is grown using conventional FIBID.

The growth of electrical contacts based on conventional FIBID and Cryo-FIBID to materials prone to ion-induced damage (2D materials, very thin nanowires, epitaxial oxides, etc.) is expected to demonstrate the usefulness of Cryo-FIBID in specific applications, which will be the subject of future work. On the other hand, the grown W-C Cryo-FIBID deposits are metallic but show a relatively-high electrical resistivity, which could hamper their application when low-resistance electrical contacts are required. Nevertheless, other precursors commonly used in FIBID for metal contacts are available and could produce the required low resistivity by Cryo-FIBID processing.

It is worth mentioning that Cryo-FIBID can be used with other ion sources, leading to different properties. The use of light-ion FIB sources such as He^+^ and Ne^+^, with larger penetration depth in matter than Ga^+^ under the same accelerating voltage, could be used for Cryo-FIBID in combination with thicker condensed precursor layers to produce deposits in the range of few-hundreds of nanometers. If thick deposits are needed, this approach would be more practical than using Ga^+^-FIB sources with higher acceleration voltage or using multiple Ga^+^-Cryo-FIBID processes. On the other hand, the use of heavy-ion FIB sources like Xe^+^, with shorter penetration depth in matter than Ga^+^ under the same accelerating voltage, could be useful towards the creation of sub-10 nm metallic deposits. Certainly, the extension of Cryo-FIBID to other FIB sources is inspiring and could give rise to applications in various fields, which merits further exploration.

## Conclusions

In the present work, we describe Cryo-FIBID (Focused Ion Beam Induced Deposition under cryogenic conditions), using a condensed precursor layer and low-dose FIB exposure. Here, the growth of metallic W-C deposits by Ga^+^-Cryo-FIBID is demonstrated. A Ga^+^ dose as low as 45 μC/cm^2^ is sufficient to grow a 30 nm-thick metal deposit, with applications in circuit editing, mask repair and the growth of electrical contacts to micro/nano-objects. As a proof of concept, we show the use of Cryo-FIBID to grow metallic contacts on a Pt-C nanowire. The ion irradiation time was less than one minute, being considerably faster than the 10-hour irradiation time needed to grow the same contacts with conventional FIBID. The ultrafast growth of materials compared to standard FIBID, together with the high lateral resolution, the low proximity effect, and the potential use of other FIB sources (with lighter or heavier ions than Ga^+^) open exciting perspectives for Cryo-FIBID and its application in various fields of nanotechnology.

## Materials and Methods

### Growth of W-C nanostructures by Ga^+^ FIBID

The W-C nanostructures have been fabricated on Si substrates either with a native (∼3 nm) SiO_2_ surface layer or a thermally-grown 300 nm-thick SiO_2_ layer. A Nova-Nanolab-200 Dual Beam instrument from FEI upgraded with a cryo-setup model PPT2000 from Quorum Technologies and equipped with a Ga^+^ focused ion beam column and an individual gas injection system (GIS) through which W(CO)_6_ gas is delivered to the process chamber, has been used. The W(CO)_6_ GIS forms a 28° angle with respect to the stage horizontal platform. The substrate cooling rate has been set to 29 °C/minute, with total cooling time from 25 °C to −100 °C of 260 seconds. Before the GIS valve is opened to deliver the precursor gas, the precursor material is heated to 55 °C. The following irradiation parameters have been used in the deposits shown in Figs 2–4: beam voltage of 30 kV, ion beam current of 1 pA, pitch of 6 nm and dwell time at each point of 200 ns. The structures shown in Figs 5 and 6, with much larger area, have been grown using a 10 pA current. After irradiation, the sample stage is heated from −100 °C to 50 °C in less than 5 minutes and stays at that temperature for 10 minutes.

### Scanning transmission electron microscopy

STEM imaging and EDS were carried out with a probe-corrected FEI Titan 60–300 operated at 300 kV and equipped with a high brightness X-FEG and a Cs CETCOR corrector for the condenser system to provide sub-angstrom probe size. The energy resolution of the EDS experiments was approximately ~125 eV.

### Electrical characterization

Current-versus-voltage electrical experiments shown in Fig. 3 (two-probe measurement) and Fig. 6 (four-probe measurement) were performed inside Dual Beam instrument using electrical microprobes from Kleindiek and a Keithley 6221 current source and a 2182 A nanovoltimeter connected to the microprobes via a chamber feedthrough. In the measurements as a function of temperature (Fig. 3b), a Physical Properties Measurement System (PPMS) by Quantum Design was used, which measures the resistance by means of a current source and a voltmeter.

## Supplementary information

Video on Cryo-FIBID processing

Supplementary Information

## Data Availability

The datasets generated and/or analyzed during the current study are available from the corresponding author on reasonable request.
