# Supplementary material for: Ultra-fast direct growth of metallic micro- and nano-structures by focused ion beam irradiation
Source: Sci Rep. 2019 Oct 1;9:14076. doi: 10.1038/s41598-019-50411-w (PMC6773749; doi:10.1038/s41598-019-50411-w)
Supplement: Supplementary file 2 — Supplementary Information [file 41598_2019_50411_MOESM2_ESM.pdf]

Ultra-fast direct growth of metallic micro- and nano-structures by focused ion beam irradiation

Rosa Córdoba[1,2,4], Pablo Orús[1,2], Stefan Strohauer[3,6], Teobaldo E. Torres[3,5], José María De Teresa[1,2,3]

[1] *Instituto de Ciencia de Materiales de Aragón (ICMA), Universidad de Zaragoza-CSIC, 50009 Zaragoza, Spain.*
[2] *Departamento de Física de la Materia Condensada, Universidad de Zaragoza, 50009 Zaragoza, Spain.*
[3] *Laboratorio de Microscopías Avanzadas (LMA) - Instituto de Nanociencia de Aragón (INA), Universidad de Zaragoza, 50018 Zaragoza, Spain.*

[4]Current address: Instituto de Ciencia Molecular, Universitat de València, Paterna, 46980 València, Spain
[5]Current address: Instituto de Nanociencia y Nanotecnología CNEA-CONICET, Centro Atómico Bariloche, Av. Bustillo 9500, 8400 San Carlos de Bariloche, Argentina
[6]Current address: Walter Schottky Institute and Physics Department, Technical University of Munich, Am Coulombwall 4, D-85748 Garching, Germany

Growth rate versus electrical resistivity in FEBID and FIBID processes. Representative literature data of growth rates and electrical resistivity of various as-grown materials used in FEBID and FIBID processing are shown in Fig. S1. This graph illustrates the need of FIBID to achieve metal contacts using typical materials such as W, Cu and Pt. Nevertheless, in both cases, FEBID and FIBID, purification methods can be used to increase the metal content and decrease the electrical resistivity.[1]

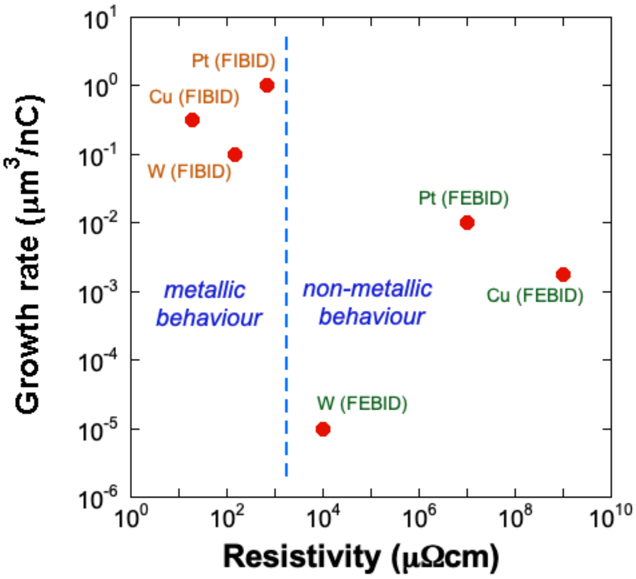

Fig. S1. Growth rate and electrical resistivity of typical as-grown FIBID and FEBID deposits used for metallic interconnects. The plot includes representative literature data of growth rates and electrical resistivity of various materials used in FEBID and FIBID processing.[2] [3] [4] [5] [6] [7] Taking into account the requirements for the growth of metal interconnects (high growth rates and low resistivity), FIBID is found to be more convenient than FEBID for this application.

$W(CO)_6$ condensed layer when the precursor is delivered at a distance of 10 mm between the gas injector and the substrate. The top-down SEM micrographs shown in Fig. S2 are informative about the homogeneity and roughness when the precursor is delivered at a distance of 10 mm with respect to the substrate. When the substrate is maintained at -100 ºC, the condensed layer is homogeneous and with low roughness, in contrast with a less homogeneous and higher roughness layer observed at -80 ºC and -60 ºC.

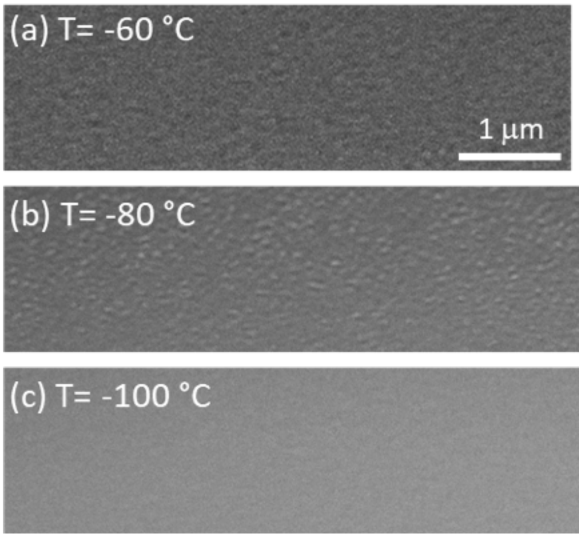

Fig. S2. SEM micrographs of the $W(CO)_6$ precursor condensed layer when the precursor is delivered at substrate temperature from -60 ºC to -100 ºC using a working distance of 10 mm between the gas injector and the substrate. A homogeneous layer of condensed $W(CO)_6$ is observed at -100 ºC. The scale bar is the same for (a), (b) and (c).

$W(CO)_6$ condensed layer when the precursor is delivered at a distance of 5 mm between the gas injector and the substrate. The SEM micrographs shown in Fig. S3 are informative about the homogeneity, roughness and presence of voids when the precursor is delivered at a short distance with respect to the substrate, 5 mm. The best results are obtained when the substrate is maintained at -80 ºC, but are not optimal compared to gas delivery when the distance to the substrate is 10 mm and the substrate is maintained at -100 ºC.

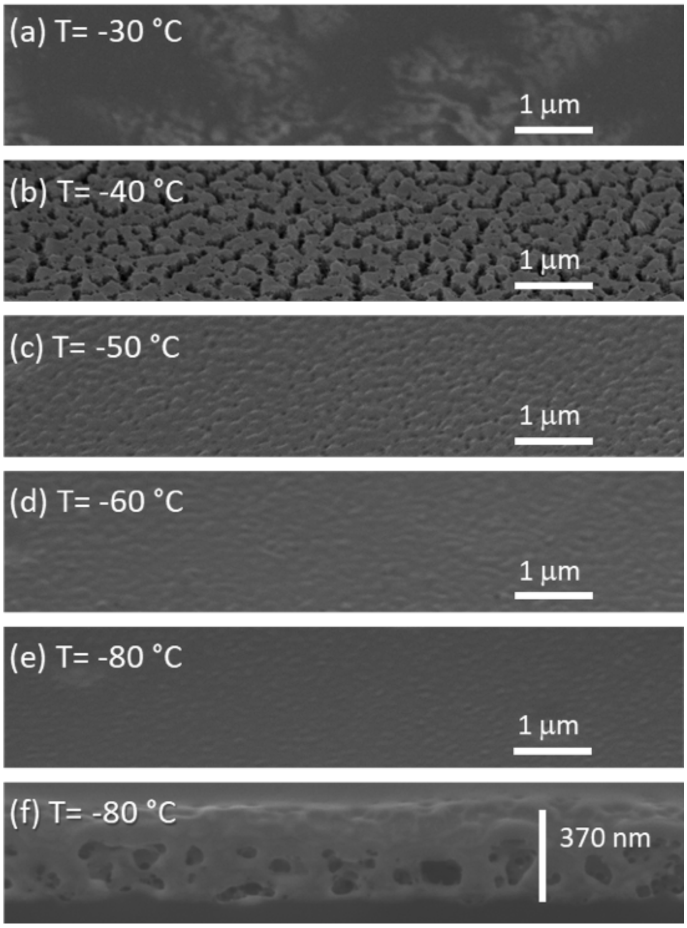

Fig. S3. SEM micrographs of the $W(CO)_6$ precursor condensed layer when the precursor is delivered at substrate temperature from -30 ºC (a) to -80 ºC (e) using a distance of 5 mm between the gas injector and the substrate. (f) a cross-sectional view of the condensed layer at -80 ºC (e). The best results are obtained at -80ºC, but roughness and voids are observed at all temperatures.

Change of porosity as a function of the ion dose. In Fig. S4, SEM micrographs of cryo-deposits grown using two different irradiation doses, one of them close to the optimal dose, are shown. Whereas the deposit exposed with 35.7 $\mu C/cm^2$ Ga^+ dose is compact and void-free, the deposit exposed with 4.21 $\mu C/cm^2$ Ga^+ dose is not compact and shows a high void density.

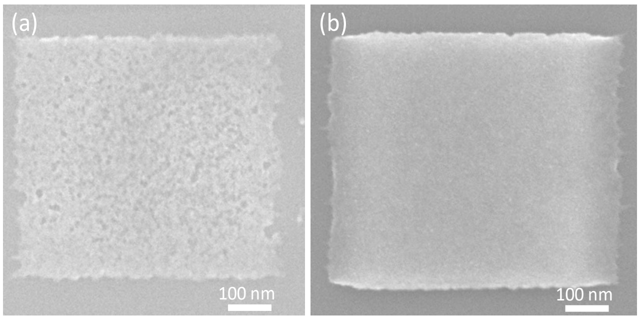

Fig. S4. SEM micrographs illustrating the change of porosity in a W-C 30 nm-thick cryo-deposit as a function of the Ga⁺ dose: (a) 4.21 μC/cm²; (b) 35.7 μC/cm².

Detailed compositional analysis by EDS of the W-C deposit grown by Cryo-FIBID at 55 μC/cm² of Ga⁺ dose. EDS experiments were carried out on small (4 nm x 4 nm) areas, from top to bottom, on the TEM lamella shown in Fig. 2 in the main manuscript. The atomic % of the detected elements is included in Fig. S5.

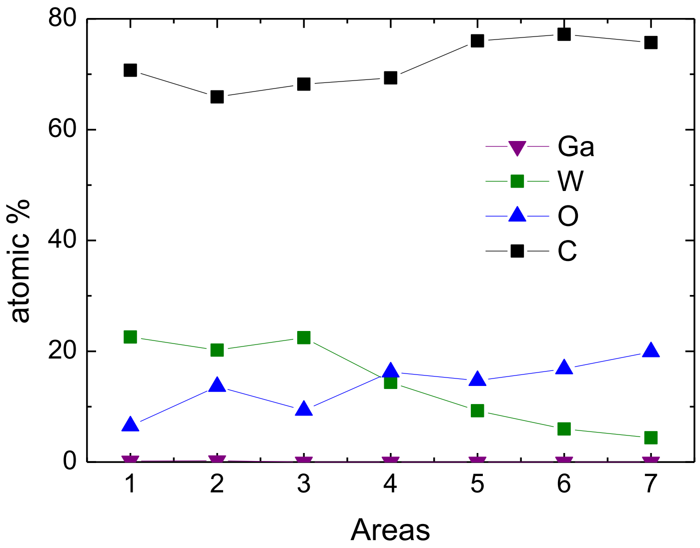

Fig. S5. Composition in atomic % of the cryo-deposit shown in Fig. 4 in the main manuscript, where the areas 1 to 7 are marked. The W content is above 20 % in the top half of the condensed layer, decreasing below 10 % in its bottom half.

On the other hand, the maximum Ga concentration can be roughly estimated by assuming that all the Ga^+ ions sent to the deposit remain inside it. Taking an ion dose of 50 μC/cm^2, and an ion penetration length of 30 nm (the same thickness as the deposit), the maximum Ga concentration expected would be 10^{20} atoms/cm^3. Taking into account that the volume of one $W(CO)_6$ molecule is 0.0343 nm^3, the number of precursor molecules per volume is 2.9 x 10^{22} cm^{-3}. This means that the ratio of the number of Ga ions to W atoms is 1/290=0.003 or 0.3%. This value is in good agreement with the EDS measurements, which indicate that the total amount of Ga in the deposit is ≤ 0.2%.

Analysis of the metallic behaviour of the W-C Cryo-FIBID deposits. Current-versus-voltage (I-V) experiments shown in Fig. S6 display a linear dependence, as expected for a metallic behaviour. From linear fits to the I-V data, the electrical resistance was extracted. The results obtained in samples grown under ion doses from 35 to 60 μC/cm^2 are displayed in Fig. 3(a) in the main manuscript.

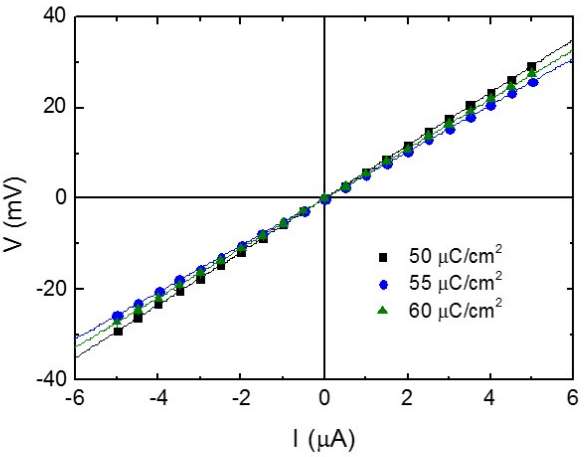

Fig. S6. Current-versus-voltage (I-V) measurements in the W-C Cryo-FIBID structures shown in Fig. 3(a) in the main manuscript, grown under various ion doses. The linear dependence indicates the metallic behaviour of these deposits.

The temperature-dependent resistance data have been analysed to determine the metallic behaviour of W-C Cryo-FIBID deposits. We have used the criterion proposed by Möbius et al.,[8] previously applied to Pt-FIBID deposits.[9] This criterion uses the calculation of the temperature-dependent (T) parameter *w*, defined as $w(T)=d(\ln\rho^{-1})/d(\ln T)$. The material is considered metallic when *w* tends to 0 as T=0 is approached. As it can be observed in Fig. S6, *w* tends to 0 for T→ 0 in the W-C wires grown by Cryo-FIBID, confirming its metallic character.

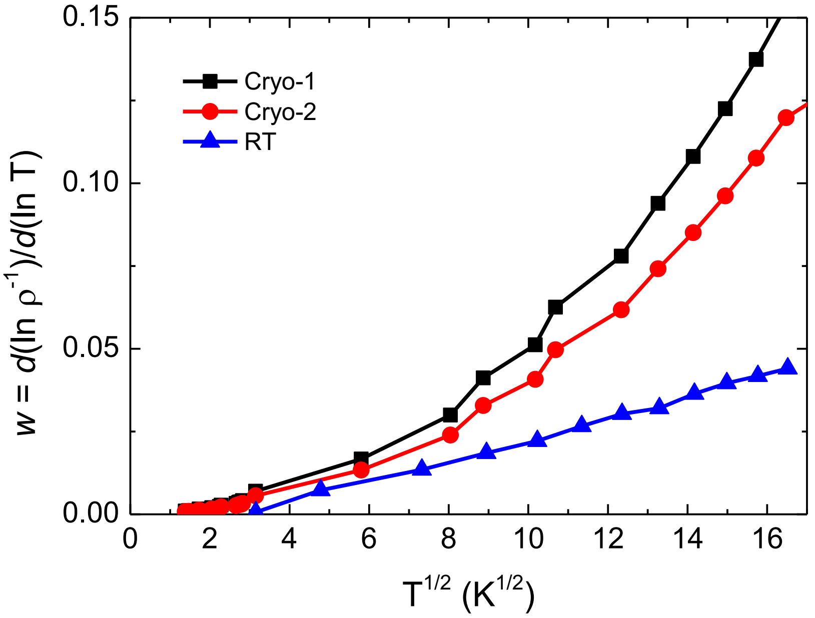

Fig. S7. Analysis of the metallic behaviour of the W-C Cryo-FIBID deposits. The parameter *w* is represented as a function of temperature for two W-C Cryo-FIBID deposits (Cryo-1,2) and for a standard W-C FIBID deposits (RT). *w* goes to zero at low temperature as expected in the case of metallic behaviour. For clarity, values of *w* for the RT deposit around and below the superconducting transition temperature (5 K) are not shown.

References

1. Perez-Roldan, M. J., Mulders, J. J. L. & Trompenaars, P. H. F. Oxygen-assisted purification of platinum structures deposited by ion and electron beam induced processes. *J. Phys. D. Appl. Phys.* **50**, 205307 (2017).
2. De Teresa, J. M. *et al.* Origin of the difference in the resistivity of as-grown focused-ion- and focused-electron-beam-induced Pt nanodeposits. *J. Nanomater.* **2009**, 936863 (2009).
3. Córdoba, R. *Functional Nanostructures Fabricated by Focused Electron/Ion Beam Induced Deposition*, Springer, 2014.
4. Gannon, T. J. *et al.* Focused ion beam induced deposition of low-resistivity copper material. *J. Vac. Sci. Technol. B Microelectron. Nanom. Struct.* **22**, 3000–3003 (2004).
5. Haverkamp, C. *et al.* A novel copper precursor for electron beam induced deposition. *Beilstein J. Nanotechnol.* **9**, 1220–1227 (2018).
6. Sadki, E. S., Ooi, S. & Hirata, K. Focused-ion-beam-induced deposition of superconducting nanowires. *Appl. Phys. Lett.* **85**, 6206–6208 (2004).
7. Li, W., Fenton, J. C., Wang, Y., Mccomb, D. W. & Warburton, P. A. Tunability of the superconductivity of tungsten films grown by focused-ion- beam direct writing. *J. Appl. Phys.* **104,** 093913 (2008).
8. Möbius, A. *et al.* Metal-insulator transition in amorphous $Si_{1-x}Ni_x$: Evidence for Mott's minimum metallic conductivity. *Phys. Rev. B - Condens. Matter Mater. Phys.* **60**, 14209–14223 (1999).
9. Fernández-Pacheco, A., De Teresa, J. M., Córdoba, R. & Ibarra, M. R. Metal-insulator transition in Pt-C nanowires grown by focused-ion-beam- induced deposition. *Phys. Rev. B* **79,** 174204 (2009).
